# Supplementary material for: Mediterranean diet and associations with the gut microbiota and pediatric-onset multiple sclerosis using trivariate analysis
Source: Commun Med (Lond). 2024 Jul 19;4:148. doi: 10.1038/s43856-024-00565-0 (PMC11271616; doi:10.1038/s43856-024-00565-0)
Supplement: Supplementary file 2 — Supplemental information [file 43856_2024_565_MOESM2_ESM.pdf]

Supplementary Information: Mediterranean Diet and Associations with the Gut Microbiota and Pediatric-Onset Multiple Sclerosis using Trivariate Analysis

Supplementary Methods.

Supplementary Fig. 1. Flow-chart showing the selection of the pediatric-onset MS cases and controls for the Diet group and Diet-Microbiota subgroup
Supplementary Fig. 2. The Diet group: The Mediterranean diet score distribution for pediatric-onset MS cases and controls
Supplementary Fig. 3. The Diet group: Correlations between the alternative Mediterranean diet score, their dietary components and specific nutrients
Supplementary Fig. 4. The Diet-microbiota subgroup: The trivariate relationships between diet, the overall microbiota (at the genus-level) and disease status (MS vs controls)
Supplementary Fig. 5. The Diet-microbiota subgroup: The trivariate relationships between diet, the overall microbiota (at the ASV-level) and disease status (MS vs controls)

Supplementary Table 1. The dietary components and scoring criteria for the alternative Mediterranean diet score.
Supplementary Table 2. Description and categorization of cohort characteristics used for the Diet group and the Diet-microbiota subgroup analyses.
Supplementary Table 3. The Diet group: The association of each component of the Mediterranean diet (g/day and specific score) with odds of MS
Supplementary Table 4. The Diet group: Association of Mediterranean diet score and nutrient intakes with odds of MS with additional adjustments for race and overweight status
Supplementary Table 5. The Diet-microbiota subgroup: Association of microbial taxa relative abundance with vitamin D supplementation among MS cases
Supplementary Table 6. The Diet-microbiota subgroup: Association of microbial taxa relative abundance with disease-modifying drug exposure among MS cases

This supplementary material has been provided by the authors to give readers additional information about their work.

Supplementary Methods.

Bioinformatics: assigning taxonomy

Amplicon sequence variants (ASVs) were assigned taxonomy using QIIME2's Naive Bayes classifier trained on SILVA taxonomy database (v. 132).[1] SILVA appends a distinct integer to the genera nomenclature to distinguish clusters that have a 94.5% sequence similarity.[2] We then removed these integer identifiers before aggregating ASVs at the genus-level, but kept them when assigning taxonomy to the ASVs. This was performed to prevent sub-genus groupings when calculating the relative abundance of each genera.

Diet-microbiota subgroup: multivariate methods

We estimated the proportion of variance (R^2) of the overall microbiota composition explained by each dietary measure, disease status, age at stool sample procurement, sex, race, overweight/obese status, and the Bristol Stool Scale group using permutational multivariate analysis of variance (PERMANOVA) via the R function *adonis* with 10,000 permutations.[3] PERMANOVA models were adjusted for race (White vs non-White), library size of the respective sample, overweight/obese status, Bristol stool scale group, age at stool sample procurement and sex and additionally adjusted for total energy intake when a dietary measure was modeled.

We then constructed a principal component (PC) index to represent the variation in the microbiota exclusively explained (constrained) by aMED, using a partial and constrained redundancy analysis via the *rda* function.[4] Redundancy analysis (RDA) performs a multivariate multiple linear regression followed by principal component analysis (PCA) of the matrix of fitted values. Like PCA, RDA computes canonical axes that are linear combinations of the outcome data (e.g. ASV-sample table) but must also be linear combinations of the explanatory variables (e.g. aMED score) that best explain the variation of the outcome data.[4] Specifically, using RDA, the variation exclusively explained by the aMED scores from the overall microbiota composition were extracted and summarized into a single principle component (PC-aMED) after removing variation explained by library size, race (White, non-White), overweight/obese status, Bristol stool scale group, age at stool sample procurement, sex and total energy intake. The PC-aMED was scaled as the weighted sums of species. This method was also applied to fiber intake to construct the PC-fiber index. We chose to evaluate fiber intake alongside the aMED score given its established influence on gut microbiota composition.[5] We tested the association between each constrained PC with MS risk using logistic regression models adjusted for library size, Bristol Stool Scale group, age and sex.

All multivariate analyses were based on the Aitcheson distance (the Euclidean distance between samples after clr-transformation) and were conducted using the R package *vegan* (v. 2.5-7).[6,7] To minimize the undue influence of potential outlying taxa in all multivariate analyses, the filtered clr-transformed taxa were winsorized to the 0.05 and 0.95 quantiles.

Supplementary Fig. 1: Flow-chart showing the selection of the pediatric-onset MS cases and controls for the Diet group and Diet-Microbiota subgroup

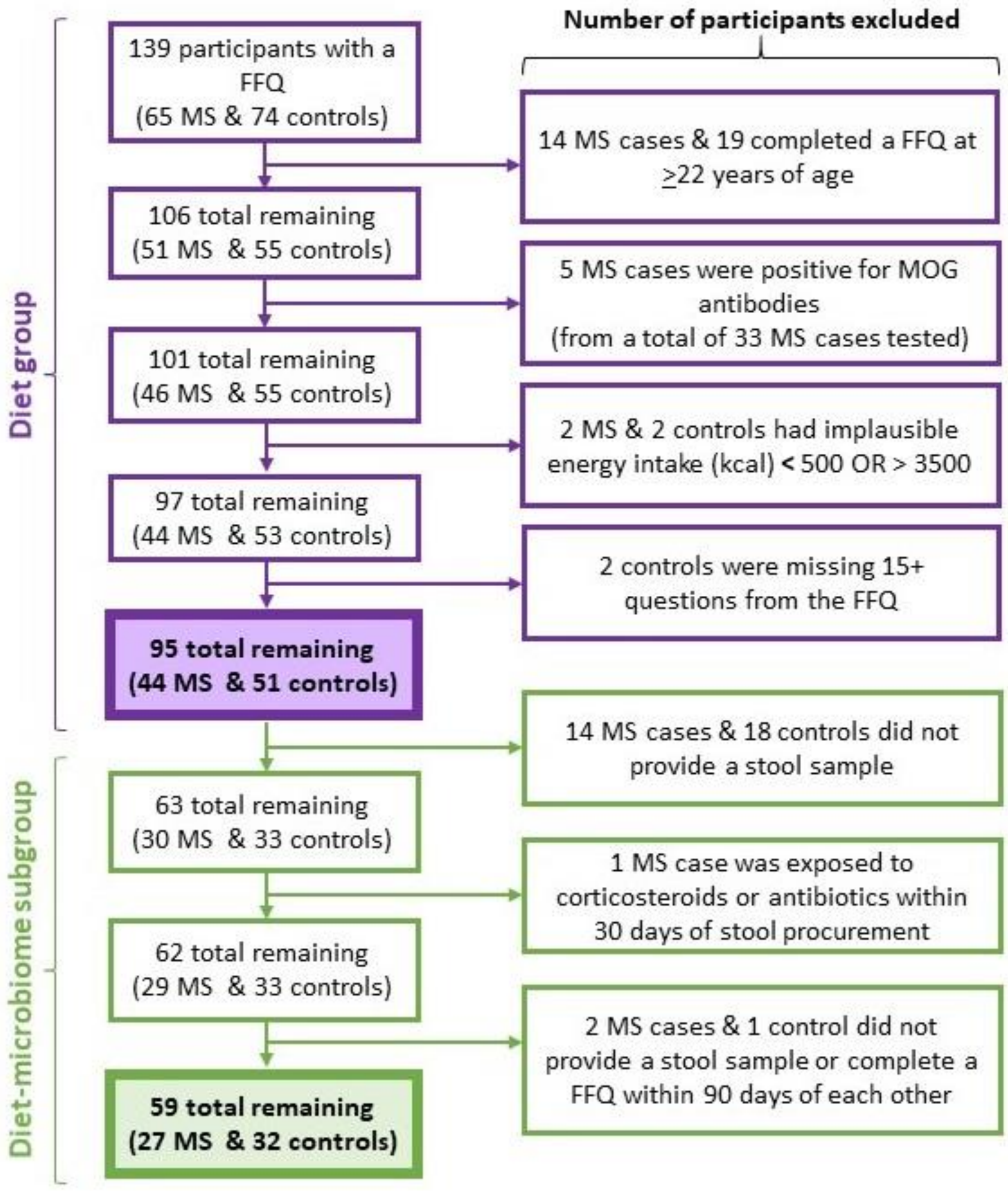

Abbreviations: MOG = Myelin oligodendrocyte glycoprotein; FFQ = food frequency questionnaire
MOG antibody-related details (including the assays used) are described elsewhere.[8]

Supplementary Fig. 2. The Diet group: The Mediterranean diet score distributions for the pediatric-onset MS cases and controls

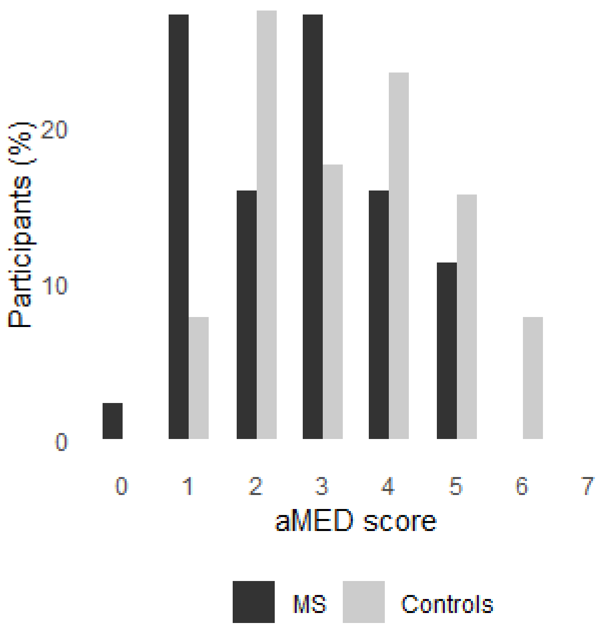

Abbreviations: aMED = alternative Mediterranean diet score

Bars represent the percent of multiple sclerosis (MS) cases or controls scoring 0 through 7 (indicating the lowest to highest resemblance to the Mediterranean diet) for aMED. Percentage were calculated with the denominator reflecting the total number of individuals in the respective groups (44 MS cases or 51 controls).

Supplementary Fig. 3. The Diet group: Correlations between the alternative Mediterranean diet score, their dietary components and specific nutrients

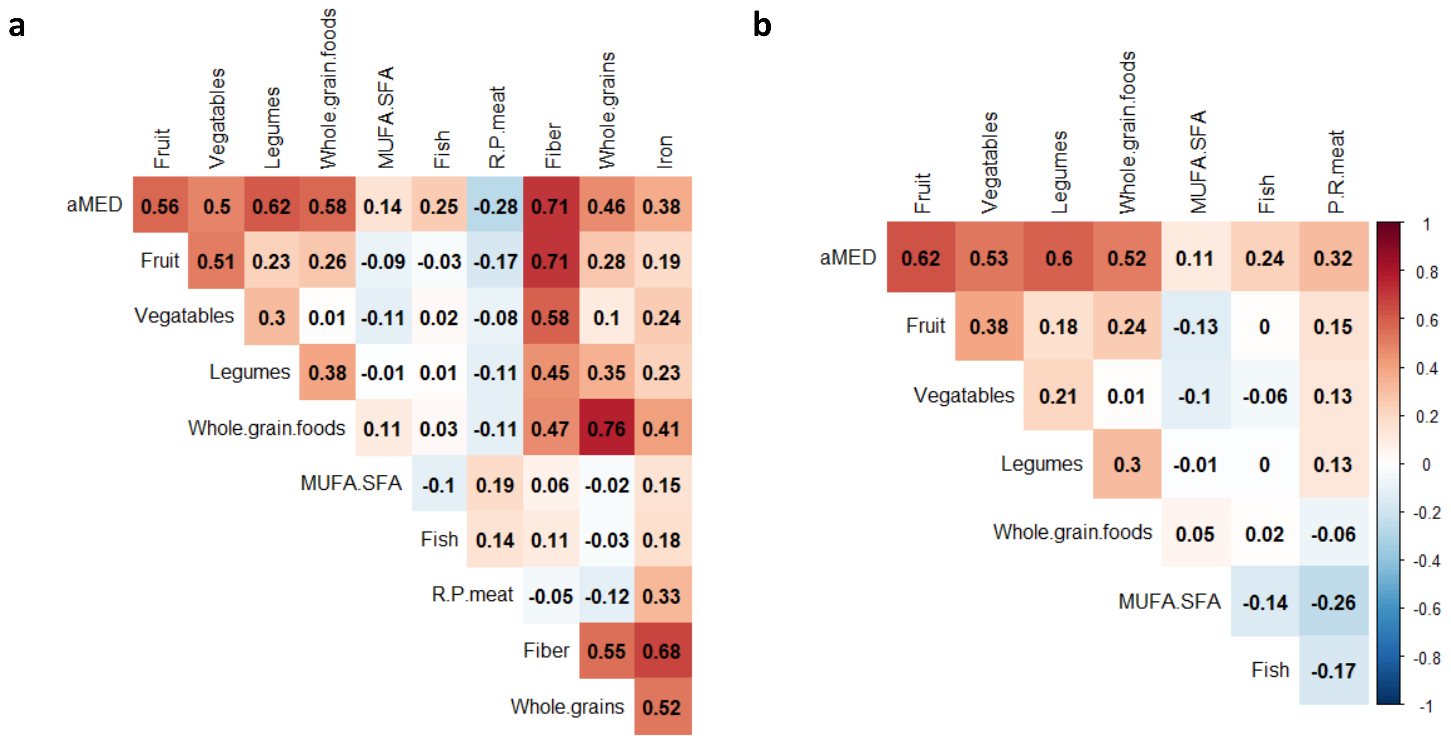

Abbreviations: aMED = alternative Mediterranean diet score; MUFA.SFA = ratio of monosaturated fatty acids to saturated fatty acids; R.P meat = red and processed meat

Pairwise spearman correlations are shown. Participants assessed include n=44 multiple sclerosis (MS) cases and n=51 controls. (a) The dietary components are in units of daily intake (grams). Nutrient intakes of fiber (g/day), whole-grains (g/day) and iron (mg/day) were also included. (b) The dietary components were dichotomized into 0 and 1 (see Supplementary Table 1 for scoring criteria). Importantly, the findings indicate that the aMED scores were not strongly correlated by any one component.

Supplementary Fig. 4. The Diet-microbiota subgroup: The trivariate relationships between diet, the overall microbiota (at the genus-level) and disease status (MS vs controls)

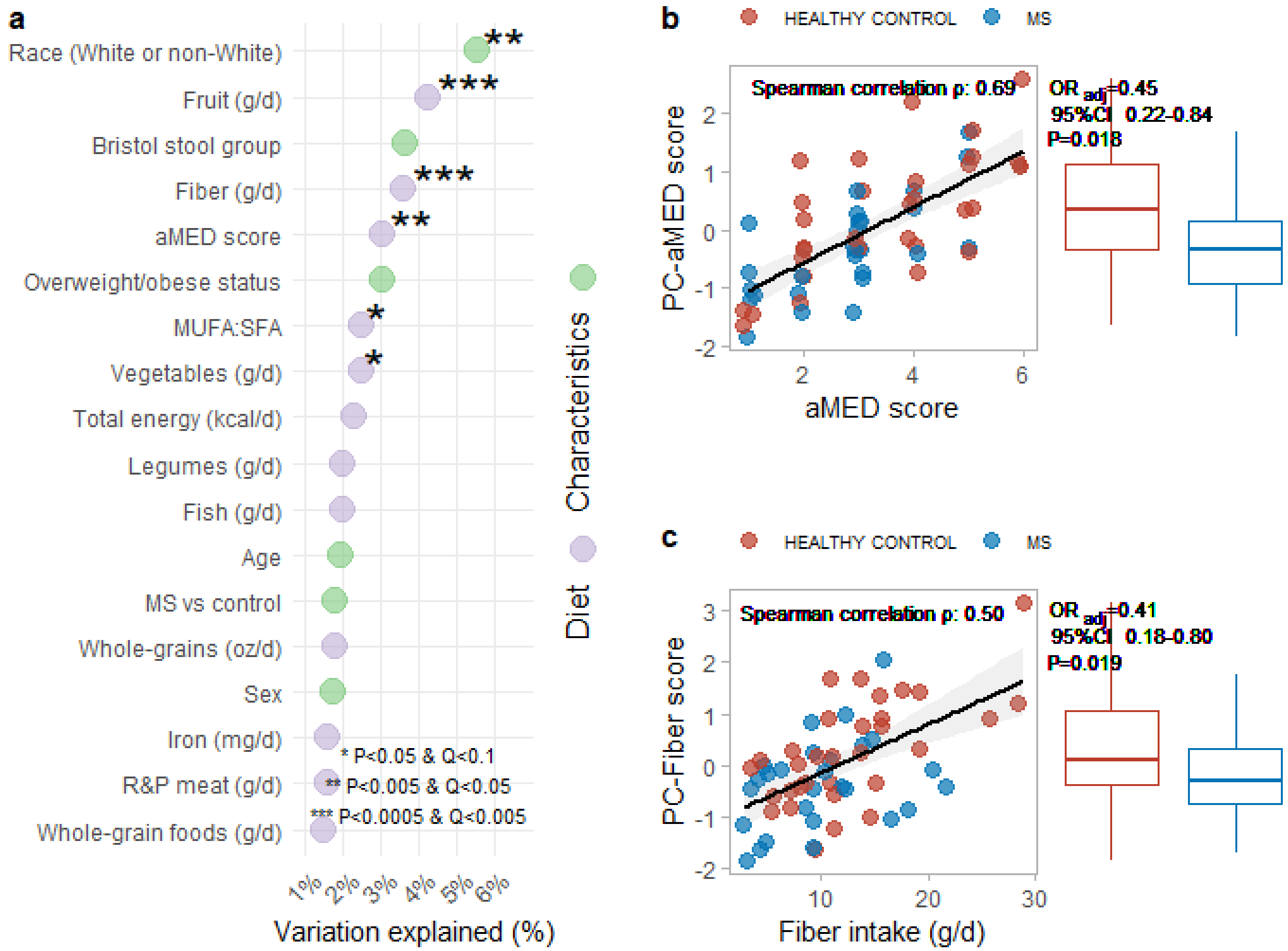

Abbreviations: MS = multiple sclerosis; aMED = alternate Mediterranean diet score; P&R meat = processed and red meat; MUFA:SFA = ratio of monounsaturated to saturated fat; OR_{adj} = adjusted odds ratio; PC-aMED = principal component of the gut microbiota composition constrained by the aMED; PC-Fiber = principal component of the gut microbiota composition constrained by fiber intake

Participants assessed include n=27 MS cases and n=32 controls. The input data for all multivariate analyses were genera relative abundance and the methods were based on the Aitcheson distance (the Euclidean distance between samples after clr-transformation) (a) Variation explained (R^2) from the overall gut microbiota using permutational multivariate analysis of variance (PERMANOVA). (b and c) The scatter plot on the left displays the correlation between the aMED score and fiber intake with PC-aMED and PC-Fiber scores, respectively. The right side is a box plot summarizing the PC scores by MS (blue) and controls (red). The association of the respective PC scores with disease status is displayed. Similar to the PERMANOVA results, PC-aMED and PC-fiber explained 3.1% and 3.6% of the gut microbiota variation, respectively, when estimated using the partial and constrained redundancy analysis. Both logistic regression models in (b) and (c) were adjusted for library size, Bristol Stool Scale group, age at the food frequency questionnaire completion and sex while any models involving diet were additionally adjusted for total energy intake.

Supplementary Fig. 5. The Diet-microbiota subgroup: The trivariate relationships between diet, the overall microbiota (at the ASV-level) and disease status (MS vs controls)

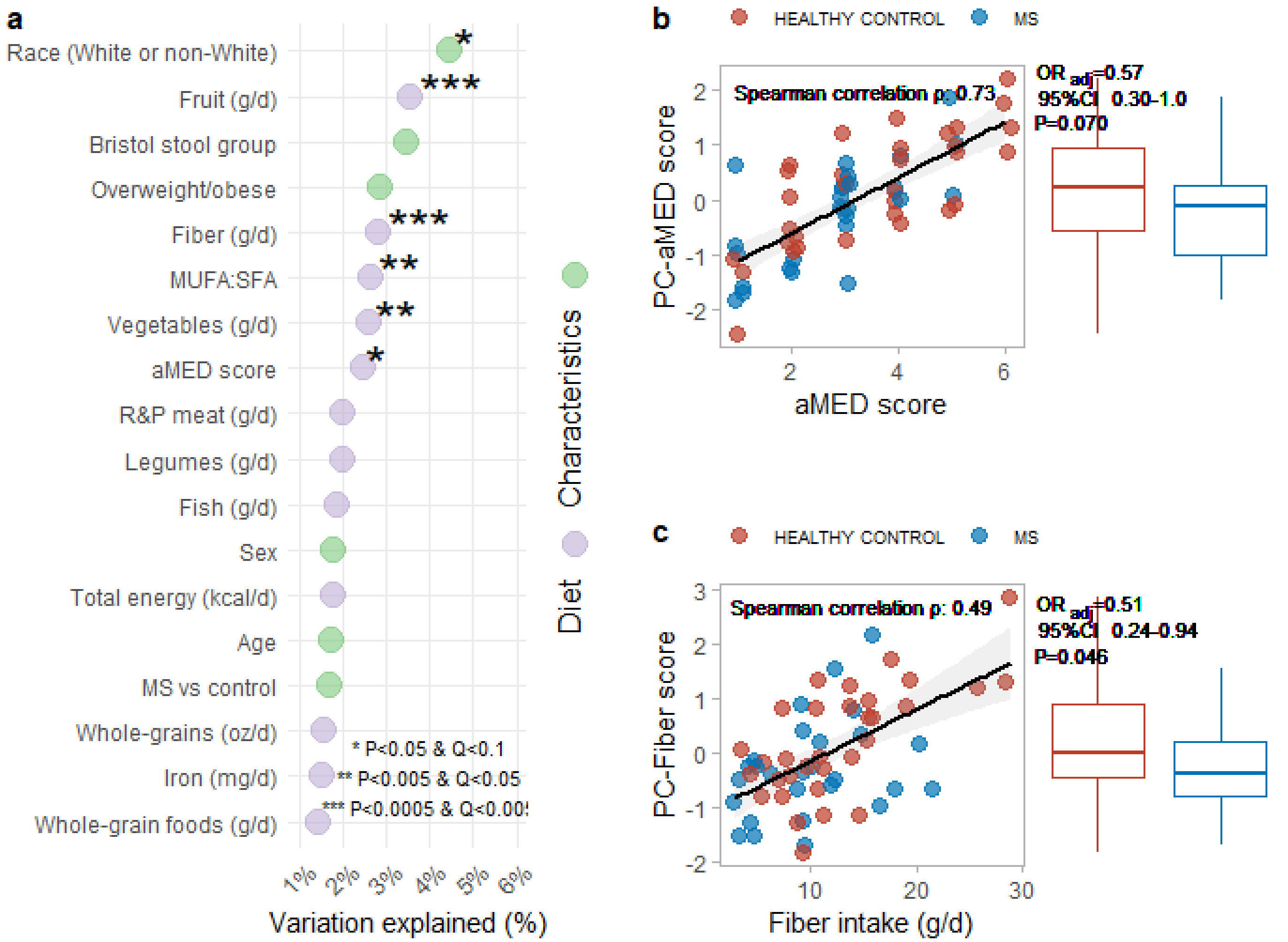

Abbreviations: MS = multiple sclerosis; aMED = alternate Mediterranean diet score; P&R meat = processed and red meat; MUFA:SFA = ratio of monounsaturated to saturated fat; NMDS = non-metric multidimensional scaling; OR_{adj} = adjusted odds ratio; PC-aMED = principal component of the gut microbiota composition constrained by the aMED; PC-Fiber = principal component of the gut microbiota composition constrained by fiber intake

Participants assessed include n=27 MS cases and n=32 controls. The input data for all multivariate analyses were amplicon sequence variants (ASVs) relative abundance and the methods were based on the Aitcheson distance (the Euclidean distance between samples after clr-transformation) (a) Variation explained (R^2) from the overall gut microbiota using permutational multivariate analysis of variance (PERMANOVA). (b and c) The scatter plot on the left displays the correlation between the aMED score and fiber intake with PC-aMED and PC-Fiber scores, respectively. The right side is a box plot summarizing the PC scores by MS (blue) and controls (red). Similar to the PERMANOVA results, PC-aMED and PC-fiber explained 2.5% and 2.6% of the gut microbiota variation, respectively, when estimated using the partial and constrained redundancy analysis. Both logistic regression models in (b) and (c) were adjusted for library size, Bristol Stool Scale group, age at the food frequency questionnaire completion and sex while any models involving diet were additionally adjusted for total energy intake.

Supplementary Table 1. The dietary components and scoring criteria for the alternative Mediterranean diet score.

aMED Components	Foods included	Criteria for 1 point[a]	Median intake[b]	
			Girls	Boys
Fruit	Apples, bananas, oranges, and any other fruit	Intake (g/day) > median of controls	145.3	91.3
Vegetables	Lettuce salad, tomatoes, green beans, peas, and any other vegetables	Intake (g/day) > median of controls	59.6	89.6
Legumes	Chili beans, pinto beans, and refried beans	Intake (g/day) > median of controls	3.1	0.0
Whole-grain foods	Cooked cereal, whole wheat bread, plain Cheerios, and grape nuts	Intake (g/day) > median of controls	24.5	14.6
Fish	Fish and fish sandwiches	Intake (g/day) > median of controls	2.0	0.0
Red and processed meat	Unprocessed red meat (defined below), hamburgers, cheeseburgers, hot dogs, corn dogs, meat balls, meat loaf, and lunch meat	Intake (g/day) < median of controls	32.0	58.5
MUFA:SFA	Ratio of monosaturated fatty acids to saturated fatty acids	Ratio > median of controls	1.0	1.0

Abbreviations: aMED = alternate Mediterranean diet score; g = grams

[a]0 points if those criteria are not met.

[b]Sex-specific median intake calculated from controls (female n=32, male n=19).

Supplementary Table 2. Description and categorization of cohort characteristics used for the Diet group and the Diet-microbiota subgroup analyses

Variable name	Variable description	Additional information (related to the analyses or further categorization of the variable, if applicable)
Sex	Male; Female (binomial).<br><br>Biological sex.	-
Age	Age at time of food frequency questionnaire (FFQ) completion and age at stool sample collection in years.	The median and interquartile range of the two ages were the same. Thus, the age at time of FFQ completion was used in all analysis except for the multivariate analyses of the microbiota, where age at time of stool procured was used instead.
Disease status	Multiple Sclerosis; Healthy Control (binomial)	-
Race	Black; Caucasian; White, Caucasian; Mexican; Hispanic & Black; Indian; Caucasian/black; South Asian; Non-White, Caucasian; Asian/Caucasian; Oriental; Pakistani; Tamil; NA (categorical)<br><br>Race was self-identified	Grouped into 3 categories (White, non-White, and unknown):<br>White = "Caucasian", "White, Caucasian", or "Caucasian."
Overweight/obese status	Participant's weight (kg) and height (m) nearest to the time of FFQ completion	Grouped into 3 categories (overweight or obese, other, and unknown:<br>Overweight or obese = Body mass index ≥85th percentiles.<br>Other = Body mass index <85th percentiles<br><br>Age- and sex-specific body mass index (BMI) percentiles were calculated from the weight and height based on the US Centers for Disease Control and Prevention growth reference curves.
Bristol Stool Scale Group	7-point ordinal scale.	Grouped into 3 categories (hard; medium; loose):<br>Hard = Types 1-2<br>Medium = Types 3-5<br>Loose = Types 6-7

Abbreviations: NA = Not available

Supplementary Table 3. The Diet group: The association of each component of the alternative Mediterranean diet (g/day and specific score[a]) with odds of MS

(a)

aMED components intake (g/day)	Median (Q1, Q3) MS cases, n=44	Controls, n=51	OR_{crude} (95% CI)	*P*	OR_{adj} (95% CI)	*P*
**Fruits**	83 (31-190)	130 (66-230)	0.80 (0.52-1.2)	0.30	0.73 (0.46-1.1)	0.17
**Vegetables**	52 (27-120)	63 (42-120)	0.98 (0.65-1.5)	0.91	0.87 (0.56-1.3)	0.54
**Legumes**	0.0 (0.0-8.8)	0.0 (0.0-15)	0.49 (0.21-0.91)	0.058	**0.45 (0.18-0.85)**	**0.041**
**Whole-grain foods**	7.3 (0.0-22)	17 (1.8-49)	0.77 (0.45-1.2)	0.28	0.76 (0.45-1.2)	0.26
**Fish**	0.0 (0.0-14)	0.0 (0.0-11)	1.2 (0.79-1.8)	0.41	1.2 (0.77-1.8)	0.47
**Red and processed meat**	52 (23-87)	43 (17-68)	1.1 (0.73-1.7)	0.65	1.2 (0.75-2.1)	0.41
**MUFA:SFA**	0.99 (0.9-1.1)	1.0 (0.92-1.1)	0.84 (0.55-1.3)	0.42	0.85 (0.54-1.3)	0.41

(b)

aMED components score (0 or 1[a])	Participants scoring 1 (%) MS=Black; Control=Gray	OR_{crude} (95% CI)	*P*	OR_{adj} (95% CI)	*P*
**Fruits**	49% / 38.6%	0.64 (0.27-1.5)	0.30	0.60 (0.24-1.4)	0.25
**Vegetables**	49% / 40.9%	0.72 (0.31-1.6)	0.43	0.57 (0.23-1.3)	0.21
**Legumes**	45.1% / 29.5%	0.51 (0.21-1.2)	0.12	0.47 (0.19-1.1)	0.090
**Whole-grain foods**	47.1% / 25%	**0.37 (0.15-0.88)**	**0.028**	**0.40 (0.16-0.97)**	**0.047**
**Fish**	47.1% / 47.7%	1.0 (0.46-2.3)	0.95	0.97 (0.42-2.2)	0.94
**Red and processed meat**	49% / 38.6%	0.63 (0.27-1.5)	0.29	0.66 (0.28-1.6)	0.35
**MUFA:SFA**	49% / 40.9%	0.72 (0.31-1.6)	0.43	0.78 (0.33-1.8)	0.56

Abbreviations: MS = multiple sclerosis; aMED = alternate Mediterranean diet score; MUFA:SFA = ratio of monosaturated fatty acids to saturated fatty acids; Q1 = 1st quartile; Q3 = 3rd quartile

[a] A score of 1 indicates a healthy amount while 0 indicates an unhealthy amount (see Supplementary Table 1 for the scoring criteria).

Results are represented as odds ratio adjusted for total energy intake, age at food frequency questionnaire completion and sex, their 95% confidence intervals and *P* values. Findings where $P < 0.05$ are in bold. (a) The table displays the summary statistics by disease status (MS and controls) and the association of a 1-standard deviation increase in the intakes of each dietary components of the aMED and odds of MS. Intakes of each dietary components were standardized into Z-scores. All Q-values were >0.32 (b) Bars represent the percent of MS cases or controls scoring 1 for each of the seven dietary components of the aMED. Percentages were calculated with the denominator reflecting the total number of individuals in the respective groups (44 MS cases or 51 controls). All Q-values were >0.36. Similar findings were observed within the smaller Diet-microbiota subgroup (results not shown).

Supplementary Table 4. The Diet group: Association of Mediterranean diet score and nutrient intakes with odds of MS with additional adjustments for race and overweight status

Dietary measure	Logistic regression models: OR_{adj} (95% CI)	
	Adjusted for total energy intake, age and sex.	Additionally adjusted for overweight/obese status and race
aMED score	**0.66 (0.47–0.90), *P*=0.011**	**0.61 (0.43–0.84), *P*=0.0040**
Fiber (g/d)	**0.87 (0.78–0.96), *P*=0.011**	**0.86 (0.76–0.95), *P*=0.0054**
Whole-grains (oz equiv./d)	0.45 (0.17–1.0), *P*=0.079	0.40 (0.14–0.94), *P*=0.058
Iron (mg/d)	**0.80 (0.63–0.96), *P*=0.030**	**0.78 (0.61–0.95), *P*=0.023**

Abbreviations: aMED = alternate Mediterranean diet score; OR_{adj} = adjusted odds ratio

Participants assessed include n=44 multiple sclerosis (MS) cases and n=51 controls. Results are represented as the association of a 1-unit increase in the respective dietary measure and odds of MS. Race was categorized as White, non-White, or unknown and overweight/obese status was categorized as overweight or obese, other, or unknown. The number of individuals with unknown race and overweight/obese status were 39 and 34 for the Diet group, respectively. Significant associations (*P*<0.05) are in bold.

Supplementary Table 5. The Diet-microbiota subgroup: Association of microbial taxa relative abundance with vitamin D supplementation among MS cases.

Genera	With vitamin D supplementation median	IQR	Without vitamin D supplementation Median	IQR	P
Methanobrevibacter	-1.5	6.5	-1.5	3.0	0.75
Variovorax	-1.2	2.0	-1.7	0.68	0.18
TM7 phylum sp. oral clone FR058	-1.5	1.5	-1.6	0.68	0.45
Eggerthella	2.2	2.4	1.6	1.7	0.23
Lactococcus	1.2	2.4	2.1	2.2	0.45
Clostridiales vadinBB60 group gut metagenome	-1.5	0.64	-1.7	1.1	0.75
Ruminococcaceae NK4A214 group	-1.5	3.7	1.8	5.2	0.25
Methanobrevibacter	-1.5	6.5	-1.5	3.0	0.75
Variovorax	-1.2	2.0	-1.7	0.68	0.18
ASVs					
Lachnoclostridium sp.	-0.53	1.6	-0.85	0.33	0.099
Ruminococcaceae sp.	-0.72	0.42	-0.74	1.60	0.87
Variovorax sp.	-0.53	2.1	-0.83	0.32	0.15
Ruminiclostridium 5 uncultured bacterium sp.	-0.47	1.7	-0.64	1.6	0.27
Caulobacter sp.	-0.70	0.42	-0.85	0.24	0.39
Eggerthella sp.	3.1	1.9	2.5	1.8	0.23
Lactococcus sp.	2.1	2.6	3.1	2.0	0.42
Ruminiclostridium 9 sp.	2.3	4.3	-0.71	2.3	0.11
Anaerofilum uncultured bacterium sp.	-0.68	1.1	-0.57	2.1	0.75
Family XIII AD3011 group uncultured bacterium sp.	-0.47	3.1	2.0	3.5	0.58
Ruminococcaceae NK4A214 group gut metagenome sp.	-0.70	0.23	2.5	5.3	0.089
Alistipes indistinctus YIT 12060 sp.	**-0.68**	**1.5**	**-0.85**	**0.35**	**0.034**

Abbreviations: IQR = Interquartile range; sp. = unknown species

We assessed differences in the relative abundance of microbial taxa between multiple sclerosis (MS) patients with (n=11) and without (n=16) vitamin D supplementation using the Wilcoxon rank sum test. The analysis was restricted to taxa previously identified as associated with MS. Our findings reveal no significant variation in these taxa with respect to vitamin D supplementation, with the notable exception of *Alistipes indistinctus YIT 12060 sp*. This specific taxon showed no correlation with the aMED score (refer to Supplementary Data 2 for details). Of note, of the controls, only one supplemented with vitamin D.

Supplementary Table 6. The Diet-microbiota subgroup: Association of microbial taxa relative abundance with disease-modifying drug exposure among MS cases.

	MS cases DMD ever exposed (n=22)		MS cases DMD naïve (n=5)		P
**Genera**	**Median**	**IQR**	**Median**	**IQR**	
Methanobrevibacter	-1.5	1.6	4.90	7.8	0.41
Variovorax	-1.5	1.7	-1.8	0.31	0.28
TM7 phylum sp. oral clone FR058	-1.5	0.75	-1.8	0.31	0.21
Eggerthella	1.7	1.5	2.6	0.99	0.61
Lactococcus	1.5	2.0	2.0	2.6	0.48
Clostridiales vadinBB60 group	**-1.5**	**1.5**	**-1.9**	**0.32**	**0.016**
Ruminococcaceae NK4A214 group	0.57	5.0	-1.6	5.5	0.28
**ASVs**					
Lachnoclostridium sp.	-0.71	0.39	-0.90	0.35	0.21
Ruminococcaceae sp.	-0.70	1.3	-0.90	0.25	0.28
Variovorax sp.	-0.69	1.7	-0.90	0.25	0.28
Ruminiclostridium 5 uncultured bacterium sp.	-0.56	1.7	-0.70	1.8	0.69
Caulobacter sp.	-0.79	0.31	-0.90	0.25	0.45
Eggerthella sp.	2.5	1.9	3.6	1.3	0.38
Lactococcus sp.	2.3	2.2	3.1	2.9	0.45
Ruminiclostridium 9 sp.	-0.50	3.2	-0.90	3.4	0.45
Anaerofilum uncultured bacterium sp.	-0.63	1.6	1.3	2.2	0.48
Family XIII AD3011 group uncultured bacterium sp.	0.19	3.2	2.8	0.66	0.28
Ruminococcaceae NK4A214 group sp.	0.28	5.1	-0.70	4.70	0.34
Alistipes indistinctus YIT 12060 sp.	**-0.69**	**1.2**	**-0.95**	**0.16**	**0.0049**

Abbreviations: MS = multiple sclerosis; DMD = disease-modifying drug; IQR = Interquartile range; sp. = unknown species

We assessed differences in the microbial taxa relative abundance between MS patients with (n=22) and without (n=5) DMD exposure using the Wilcoxon rank sum test. The analysis was restricted to taxa previously identified as associated with MS. Among these taxa, only *Alistipes indistinctus YIT 12060 sp*. and the *Clostridiales vadinBB60 group* exhibited evidence of a difference in relative abundance relative to DMD status. *Alistipes indistinctus YIT 12060 sp*. showed no correlation with the aMED score, while the *Clostridiales vadinBB60 group* did (details in Supplementary Data 2). However, MS cases exposed to a DMD demonstrated a higher relative abundance of the *Clostridiales vadinBB60 group* compared to those without DMD exposure. Therefore, DMD exposure did not appear to explain the depletion of the *Clostridiales vadinBB60* group observed in MS cases compared to controls.

Supplementary References

1. Quast C, Pruesse E, Yilmaz P, et al. The SILVA ribosomal RNA gene database project: improved data processing and web-based tools. *Nucleic Acids Res.* 2013;41(Database issue):D590-596.
2. Henderson G, Yilmaz P, Kumar S, et al. Improved taxonomic assignment of rumen bacterial 16S rRNA sequences using a revised SILVA taxonomic framework. *PeerJ.* 2019;7:e6496.
3. Anderson MJ. A new method for non-parametric multivariate analysis of variance. *Austral ecology.* 2001;26(1):32-46.
4. Legendre P, Legendre L. *Numerical ecology.* Elsevier; 2012.
5. Sanchez JMS, DePaula-Silva AB, Libbey JE, Fujinami RS. Role of diet in regulating the gut microbiota and multiple sclerosis. *Clin Immunol.* 2022;235:108379.
6. Aitchison J, Barceló-Vidal C, Martín-Fernández JA, Pawlowsky-Glahn V. Logratio analysis and compositional distance. *Mathematical geology.* 2000;32(3):271-275.
7. Borcard D, Gillet F, Legendre P. *Numerical ecology with R.* Vol 2: Springer; 2011.
8. Waters P, Fadda G, Woodhall M, et al. Serial Anti-Myelin Oligodendrocyte Glycoprotein Antibody Analyses and Outcomes in Children With Demyelinating Syndromes. *JAMA Neurol.* 2020;77(1):82-93.
