# Supplementary material for: Mediterranean diet and associations with the gut microbiota and pediatric-onset multiple sclerosis using trivariate analysis
Source: Commun Med (Lond). 2024 Jul 19;4:148. doi: 10.1038/s43856-024-00565-0 (PMC11271616; doi:10.1038/s43856-024-00565-0)
Supplement: Supplementary file 3 — Description of Additional Supplementary Files [file 43856_2024_565_MOESM3_ESM.pdf]

Description of Additional Supplementary Information.

File name: Supplementary Data 1

File Description: The Diet-microbiota subgroup: ASV- and genus-level relative abundance differences in the gut microbial communities between MS cases and controls

Abbreviations: ASV = amplicon sequence variant; Q1 = 1st quartile; Q3 = 3rd quartile a. The confidence level indicates the percent of the nucleotide bases in the sequence that match the taxonomy. Summary statistics and differences of genera and ASVs abundances by disease status (MS cases vs controls) are represented as odds ratios (OR) adjusted for read depth, Bristol Stool Scale group, age (in years) at food-screener completion and sex, their 95% CIs, and P. All Q value were >0.68. An OR<1 implies a 'protective' ssociation, meaning that a 1-standard deviation increase in the taxa is associated with a lower odds of MS, and an OR>1 implies a 'detrimental' association, meaning that a 1- standard deviation increase in the taxa is associated with higher odds of MS. All genera and ASVs original raw counts were centered-log ratio (clr) transformed (that is, divided by the sample geometric mean and then log transformed) after adding a pseudo-count of 0.5 (to avoid taking the log of 0). Thus, taxa abundances higher and lower than the geometric mean have positive and negative values, respectively. Taxa were standardized to Z-scores when modeled in relation to MS risk. Only taxa with a significance of P<0.10 are listed and taxa with P<0.05 are in bold.

File name: Supplementary Data 2

File Description: Abbreviations: aMED = alternate Mediterranean diet score; MUFA:SFA = ratio of monounsaturated to saturated fat; sp. = an unnamed species. The association of each taxa with disease status (outcome variable) was represented as standardized log odds ratios. The 7 genera and the 12 ASVs that were associated with MS risk (P <0.05) were displayed. The association of each dietary measure with taxa (the outcome variable) were represented as standardized beta coefficients. All results were adjusted for read depth, Bristol Stool Scale group, age at the foodscreener completion and sex. Beta coefficients were additionally adjusted for total energy intake. Dietary measures and taxa were standardized to Z-scores only when modeled as an independent variable. Significant associations are in bold and strong significant associations (absolute beta ≥0.70) are represented with a green cell and thick outside borders.

File name: Supplementary Data 3

File Description: Raw values of the aMED score, nutrient intakes, and microbial taxa underlying all figures and tables.
