# Supplementary material for: Mediterranean diet and associations with the gut microbiota and pediatric-onset multiple sclerosis using trivariate analysis
Source: Commun Med (Lond). 2024 Jul 19;4:148. doi: 10.1038/s43856-024-00565-0 (PMC11271616; doi:10.1038/s43856-024-00565-0)
Supplement: Supplementary file 7 — Reporting Summary [file 43856_2024_565_MOESM7_ESM.pdf]

nature portfolio

Corresponding author(s): Helen Tremlett
Last updated by author(s): Mar 14, 2024

Reporting Summary

Nature Portfolio wishes to improve the reproducibility of the work that we publish. This form provides structure for consistency and transparency in reporting. For further information on Nature Portfolio policies, see our Editorial Policies and the Editorial Policy Checklist.

Statistics

For all statistical analyses, confirm that the following items are present in the figure legend, table legend, main text, or Methods section.

n/a	Confirmed	
☐	☒	The exact sample size (*n*) for each experimental group/condition, given as a discrete number and unit of measurement
☐	☒	A statement on whether measurements were taken from distinct samples or whether the same sample was measured repeatedly
☐	☒	The statistical test(s) used AND whether they are one- or two-sided<br>*Only common tests should be described solely by name; describe more complex techniques in the Methods section.*
☐	☒	A description of all covariates tested
☐	☒	A description of any assumptions or corrections, such as tests of normality and adjustment for multiple comparisons
☐	☒	A full description of the statistical parameters including central tendency (e.g. means) or other basic estimates (e.g. regression coefficient) AND variation (e.g. standard deviation) or associated estimates of uncertainty (e.g. confidence intervals)
☐	☒	For null hypothesis testing, the test statistic (e.g. F, t, r) with confidence intervals, effect sizes, degrees of freedom and P value noted<br>*Give P values as exact values whenever suitable.*
☒	☐	For Bayesian analysis, information on the choice of priors and Markov chain Monte Carlo settings
☒	☐	For hierarchical and complex designs, identification of the appropriate level for tests and full reporting of outcomes
☐	☒	Estimates of effect sizes (e.g. Cohen's *d*, Pearson's *r*), indicating how they were calculated

Our web collection on statistics for biologists contains articles on many of the points above.

Software and code

Policy information about availability of computer code

Data collection	Raw DNA sequences were obtained from the MiSeq instrument using it's intrinsic on-instrument software: Real-time analysis (RTA) software.
Data analysis	QIIME 2 (v.2019.4) and Deblur (v.1.1.0) via QIIME 2, were used to processes the microbiome data; R package LULU (v. 0.1.0) was used to curate the microbiota amplicon sequence variants; R package vegan (v. 2.5-7) was used to perform multivariate analyses; R package stats and robustbase (v. 0.93-9) was used to model microbiota data; R package mediation (v. 4.5.0) was used to perform mediation analysis; All statistical analyses were conducted using R (v. 4.0.4).

For manuscripts utilizing custom algorithms or software that are central to the research but not yet described in published literature, software must be made available to editors and reviewers. We strongly encourage code deposition in a community repository (e.g. GitHub). See the Nature Portfolio guidelines for submitting code & software for further information.

Data

Policy information about availability of data

All manuscripts must include a data availability statement. This statement should provide the following information, where applicable:
- Accession codes, unique identifiers, or web links for publicly available datasets
- A description of any restrictions on data availability
- For clinical datasets or third party data, please ensure that the statement adheres to our policy

All 59 raw amplicon sequencing data associated with this study are available under the National Center for Biotechnology Information (NCBI) BioProject accession

number PRJNA1000059. Other datasets generated and analyzed during the current study are available from the study team (contact the corresponding author, H.T.), upon reasonable request along with a proposed rigorous research question. The complete data are not publicly available due to data protection and confidentiality requirements.

Research involving human participants, their data, or biological material

Policy information about studies with human participants or human data. See also policy information about sex, gender (identity/presentation), and sexual orientation and race, ethnicity and racism.

Reporting on sex and gender

Participant characteristics, captured via questionnaires or standardized forms completed by site investigators and/or participants, included sex. Sex (biological attribute) was considered in the study design as captured using standardized form completed by the participants. Females predominated, representing 75% of MS cases and 63% of controls. 33/11 of females/males had multiple sclerosis and 32/19 females/males were controls. All statistical models were adjusted for sex. Median age of females and males were 16.7 and 15.6, respectively. For females/males, number (%) of obese or overweight was 11(16%)/4(13%), number (%) of White race was 20(31%)/6(20%).

Reporting on race, ethnicity, or other socially relevant groupings

Race was self-identified and captured via questionnaires or standardized forms. Participants identified themselves as: Black, Caucasian, "White, Caucasian", Mexican, Hispanic & Black, Indian, Caucasian/black, South Asian, "Non-White, Caucasian", Asian/Caucasian, Oriental, Pakistani, and Tamil. These races were grouped into 3 categories (White, non-White, and unknown): White = "Caucasian", "White, Caucasian", or "Caucasian." We recognize that race is a social construct and structural racism has adverse outcomes on many health indicators. We adjusted the main diet-related findings for race.

Population characteristics

Of the 95 total participants, 65 were female (biological sex). At the time of food-frequency questionnaire completion, the median age was 16.6 years old, 15 were overweight or obese, 15 had an atopy-related conditions (asthma, dermatitis, psoriasis, and acne), 2 had smoked (ever). Twenty six (59%) of MS cases were ever exposed to a disease-modifying drug.

Recruitment

MS cases and controls were enrolled through the Canadian Paediatric Demyelinating Disease Network study, which included participants from seven sites—six Canadian and one USA.

Ethics oversight

The research ethics boards at each institution approved the study, including the University of British Columbia, Children's Hospital of Philadelphia, University of Pennsylvania, University of Manitoba, and University of Toronto.

Note that full information on the approval of the study protocol must also be provided in the manuscript.

Field-specific reporting

Please select the one below that is the best fit for your research. If you are not sure, read the appropriate sections before making your selection.

☒ Life sciences ☐ Behavioural & social sciences ☐ Ecological, evolutionary & environmental sciences

For a reference copy of the document with all sections, see nature.com/documents/nr-reporting-summary-flat.pdf

Life sciences study design

All studies must disclose on these points even when the disclosure is negative.

Sample size

Based on our gut microbiome pilot study (PMID: 27176462) in pediatric MS (relative to healthy controls) where we identified 14 Phyla, we estimate at least 14 cases and 42 healthy controls are needed to detect whether there are any compositional differences at the Phylum level between the two groups using a likelihood ratio test with an $\alpha = 0.05$, and $1 - \beta \geq 0.80$. Given that we have almost double the number of cases (n=27) and a similar number of controls (n=32), we do not expect power to be an issue for our microbiota-related analyses. Given that pediatric-onset MS is rare, our sample size was modest in size.

Data exclusions

Excluded: persons not meeting the inclusion criteria. Inclusion criteria: Enrolled participants who had completed a Block Kids Food Screener (BKFS) before reaching 22 years of age. The 'Diet-microbiota subgroup' included only those participants from the Diet group who provided a stool sample within +/-90 days of completing the FFQ (representing a time period during which major dietary patterns were observably relatively stable), and who had not taken antibiotics or corticosteroids before 30 days of stool collection. Other exclusions: Participants with 15 or more missing answers on the FFQ or with implausibly low or high total energy intake (<500 or >3500 kcal/day) were excluded.

Replication

NA

Randomization

NA

Blinding

NA

Reporting for specific materials, systems and methods

We require information from authors about some types of materials, experimental systems and methods used in many studies. Here, indicate whether each material, system or method listed is relevant to your study. If you are not sure if a list item applies to your research, read the appropriate section before selecting a response.

Materials & experimental systems

n/a	Involved in the study
☒	☐ Antibodies
☒	☐ Eukaryotic cell lines
☒	☐ Palaeontology and archaeology
☒	☐ Animals and other organisms
☒	☐ Clinical data
☒	☐ Dual use research of concern
☒	☐ Plants

Methods

n/a	Involved in the study
☒	☐ ChIP-seq
☒	☐ Flow cytometry
☒	☐ MRI-based neuroimaging
